# Demographic and Clinical Outcomes of Brazilian Patients With Stage III or IV Non–Small-Cell Lung Cancer: Real-World Evidence Study on the Basis of Deterministic Linkage Approach

**DOI:** 10.1200/GO.21.00228

**Published:** 2021-10-05

**Authors:** Carlos Gil Ferreira, Marcia Datz Abadi, Paula de Mendonça Batista, Fernando Brandão Serra, Rodrigo Buzzatti Peixoto, Lucas Miyake Okumura, Erica Regina Cerqueira

**Affiliations:** ^1^ Instituto Oncoclínicas, São Paulo, Brazil; ^2^ MSD Brazil, São Paulo, Brazil; ^3^ Techtrials, São Paulo, Brazil

## Abstract

Non–small-cell lung cancer (NSCLC) is the most common type of lung cancer and accounts for 80%-90% of the cases. In Brazil, between 2018 and 2019, lung cancer was ranked as the second most frequent cancer among men and the fourth among women. The primary objectives were to describe the journey and survival rates of patients with advanced NSCLC treated in the Brazilian private health care system (HCS).

**MATERIALS AND METHODS:**

A retrospective cohort study was based on the search in administrative databases to analyze the Brazilian private HCS. Patients with advanced NSCLC diagnosed between 2011 and 2016 were included. The data on demographics, cancer-related information, treatment-related information, and resources used were collected. Survival analyses were performed using the semiparametric Kaplan-Meier method to assess mortality by NSCLC stage, with NSCLC diagnosis as the index date.

**RESULTS:**

A total of 5,016 patients were included. Most patients were between 60 and 69 years old (33.6%) and had completed elementary school (52.2%). There was a greater proportion of men (58.1% *v* 41.9%), and the majority of patients had stage IV NSCLC (67%). It took an average of 31 days, from the first consultation, to have diagnosis. In 44% of the cases, a clinical oncologist was the first specialist in the HCS that the patient was referred to. After the diagnosis, the median time to start of treatment was 35 days. Chemotherapy alone was the most common treatment regimen (32%). The median overall survival was 11.5 months and 6 months for stage II and IV NSCLC, respectively.

**CONCLUSION:**

This study provides contemporary data on stage III and IV NSCLC in private health care in Brazil, which has shown a high rate of metastatic disease diagnoses, high health care–related costs, and low survival rates.

## INTRODUCTION

According to the WHO, globally, about one in six deaths is due to cancer. In 2018, one of the most common neoplasms was lung cancer, accounting for 2.09 million cases and 1.76 million deaths.^1^ In Brazil, between 2018 and 2019, 18,740 new cases of lung cancer were estimated among men and 12,530 cases among women, with non–small-cell lung cancer (NSCLC) ranked as the second and fourth most frequent neoplasm in these populations, respectively.^2^

**CONTEXT**

**Key Objective**
How is the journey of a patient with advanced non–small-cell lung cancer in Brazilian private health care system (HCS)?
**Knowledge Generated**
Patients with non–small-cell lung cancer (NSCLC) are more commonly diagnosed by a clinical oncologist on stage IV, and it took approximately 2 months from the first consultation to start of treatment.
Patients with advanced NSCLC are more commonly treated with chemotherapy alone in Brazilian private HCS, and these patients show low survival rates, similar to the public HCS.
**Relevance**
Knowledge in advanced NSCLC patient's journey allows the perception of a necessity for improving NSCLC care by stimulating earlier tumor detection and multidisciplinary approach and by providing access to molecular testing, chemotherapy, targeted therapies, and immunotherapies

NSCLC is the most common lung cancer and accounts for 80%-90% of all lung cancers. Squamous cell carcinoma, adenocarcinoma, and large-cell carcinoma are all subtypes of NSCLC, among which adenocarcinoma is the most common one.^3,4^ Cetin et al^5^ showed 1-year survival rates of 29.1% for patients with stage IV bronchioloalveolar adenocarcinoma and 12.8% for those with large-cell tumors.^5^ The age-standardized 5-year survival rate in Brazil is 18%, which is consistent with global rates ranging from 10% to 20%.^6^ In Brazil, from 1979 to 2004, the lung cancer mortality increased from 10.6 to 31.1 deaths per 100,000 population for men and from 3.0 to 5.4 deaths per 100,000 population for women.^7^ Although more curative-intent treatment and improved relative survival for NSCLC were seen over time, these benefits were less evident among elderly people; also, differences were less marked among the elderly; also, disparities in treatments and relative survival between age groups were widening for stages III and IV in this group of patients.^8^

Regarding the availability of diagnostic procedures, in a 2005 survey, the numbers of computed tomography scanners per 1,000,000 population were 4.9 and 30.8 in the public and private health care systems (HCSs) in Brazil, respectively.^9^ These numbers highlight the difficulties in access to an adequate diagnostic evaluation in the public HCS, whereas the numbers for private health care facilities are similar to those in developed countries, such as the United States and Japan (31.5 and 32.2, respectively).^10^ In Brazil, 2010 was the year that positron emission tomography was approved for lung cancer staging in the private health care setting; however, the public HCS incorporated the technology only in 2014.^10^

According to National Health Research, performed by Brazilian Institute of Geography and Statistics, 71.1% of the Brazilian population depends on the public healthcare system Sistema Único de Saúde to have access to health services, whereas 28.4% of people have access to the private health service (PNS 2013).^11^ This inequality in access to health is proportional to access to drug products and molecular testing between public and private HCSs. Approximately two thirds of the epidermal growth factor receptor mutation tests are performed in the private sector for lung cancer, and only one third is performed in public health care institutions.^12^ In addition, standard third-generation chemotherapy agents were only introduced in the public HCS in the late 2000s and pemetrexed is still unavailable. Targeted therapies, such as monoclonal antibodies (bevacizumab) and first-generation epidermal growth factor receptor tyrosine kinase inhibitors, are available only for patients with private health care coverage.^10^

The primary objectives of this study were to characterize patients with NSCLC journey in the private health care and to explore the demographics and survival of patients with advanced NSCLC in the Brazilian private HCS. The journey of patients can be defined as the ongoing sequence of care procedures that a patient follows from the point of access into the HCS, continuing toward diagnosis and care and until the completion of outpatient care.^13^

To achieve these aims, we used database linkage, also known as record linkage at the individual level of the patient, which is the matching of separate database records, combining information from each database into a single observation unit. The challenge is to perform matching so that available data from the same person are not duplicated, whereas information from different people is not combined.^14^ Record linkage from medical databases such as electronic health records, health insurance company claims, and patient-generated data is becoming increasingly important for delivering high-quality and high-value health care, conducting valid and generalizable research, and evaluating health care policy. Database linkage can help create comprehensive, longitudinal data sets with information on patients' conditions and treatments over time.^15^

Database linkage stems from public administration disciplines and is very common in social security sciences and public administration. In the past few decades, linkage methods were adopted in the health sciences (epidemiology and public health) to solve the problem of not having integrated information about the population.^16^ Nowadays, linkage is a discipline in the public health graduate program in Brazil,^17^ and many authors have been using it to answer questions about many clinical conditions,^16,17^ such as cancer and tuberculosis. Using linkage methods is not a common practice in countries with integrated data such as the United Kingdom or even the US Medicare system.

## MATERIALS AND METHODS

### Study Design and Patients

A retrospective cohort study on the basis of administrative databases was conducted for an analysis of the real-world data on the Brazilian private HCS. We followed the International Society for Pharmacoeconomics and Outcomes Research real-world evidence analysis guidelines.^18^

Patients with advanced NSCLC diagnosed between 2011 and 2016 were included. The inclusion criteria are as follows: (1) lung cancer diagnosis (International Classification of Diseases [ICD]-10 code C34, stage III or IV) and (2) compatible histologic subtype for NSCLC, such as adenocarcinoma, squamous cell, nonsquamous cell, and others. We excluded patients who did not have histologic status or ICD code registries and patients < 18 years age, as their condition is unlikely to be due to lung cancer.

### Institutional Review Board Approval and Consent to Participate

This study does not require Institutional Review Board (IRB) approval since it is based on a public database and patients cannot be identified (anonymity is guaranteed in such databases, as no names, social security number, or other personal identification code are available [Public database searches do not require IRB approval and are based on the following legislation: Brazilian Ministry of Health, National Council on Ethics and Research, Resolution No. 510 dated April 7, 2016]).^19^

### Data Collection

Three different databases were used as primary data sources: Instituto Nacional do Câncer (INCA)/Registro Hospitalar do Câncer (RHC),^20^ Fundação Oncocentro de São Paulo (FOSP),^21^ and Agência Nacional de Saúde Suplementar (ANS)^22^ databases. These are publicly available databases comprising data from the private HCS, and they were linked by a method called deterministic record linkage.^23,24^

### Resources

Techtrials database was used to link the data of the three databases (ANS, INCA/RHC, and FOSP) to determine the number of different procedures that were performed throughout the study period and the costs for each one. The costs during hospital admission include hospital-related procedures.

The variables of interest in the study were classified into the following four groups:

1. Group 1: demographic variables (age, sex, and education). These variables were presented in proportions (percentages);
2. Group 2: variables related to the disease (stage III or IV, histologic subtype of cancer, time elapsed between the first consultation and the diagnosis of cancer, and time elapsed between the diagnosis of cancer and the beginning of treatment). The first two variables were presented in the form of absolute numbers followed by the proportions (percentages); time variables were presented as medians;
3. Group 3: type of treatment (chemotherapy, radiotherapy, and surgery). Absolute numbers were presented, followed by the proportions (percentages) of each type of treatment (separated—eg, only chemotherapy—or associated—eg, chemotherapy with radiotherapy);
4. Group 4: Resources used (duration, cost, and type of resource used). Hospital interventions and procedures (eg, chemotherapy, radiotherapy, and the main manipulations performed, in addition to the total daily cost) were included in this group. The duration of the procedure or length of stay was presented as an average number of days, accompanied by the respective minimum and maximum variations; the costs were presented in the form of average values (the US dollars [USD] to Brazilian Reais exchange rate was set at USD 1 = R$ 4,202), also accompanied by the minimum and maximum variations.

### Data Analysis

#### Database linkage and quality assurance.

Deterministic and probabilistic are the two approaches for linkage. We are using the deterministic linkage because we can match variables in a direct way (male patient > 18 years age and ICD-10 code C34 between databases, diagnosed in October 2014 in São Paulo), which is not the case with the probabilistic approach. The database entries were selected on the basis of age (date of birth–based calculation), sex, primary tumor ICD code, year of diagnosis, reasons for not being treated, place of residence (state), education level and place of birth (state), and linkage following the deterministic approach.

#### Representativeness of the NSCLC sample from 2011 to 2016.

To ensure the representativeness of our sample after the deterministic linkage, five assumptions were made on the basis of clinical and epidemiologic rationale: (1) the final number of unique identification codes should not be greater than the estimated lung cancer and NSCLC incidence and prevalence in Brazil; (2) the proportion of men with NSCLC should be greater than that of women; (3) survival in the present project should be lower to similar when compared with developed countries, and the mortality verified in this project was slightly higher than previous reports; (4) there should be a higher mortality rate in stage IV NSCLC (in comparison with stage III NSCLC) and higher risk of deaths in older patients; and (5) demographically, the sample should be homogeneous throughout the years, especially because it was different throughout the years.

#### Survival analyses.

Survival analyses were performed using the semiparametric Kaplan-Meier method to assess mortality by NSCLC stage, considering diagnosis as the index date. The purpose of this type of analysis is to estimate the survival time of patients with NSCLC in each of the two stages of interest and to compare them. The survival curves were presented in months, in the form of a graph.

### Other Statistical Methods

To compare proportions, the chi-square test was used and *P* values < .05 were considered significant. Using a 5% alpha, this study, which is composed of 5,000 patients, can be considered a powered sample (power > 80%) when testing the hypothesis that a 10-month median survival in the previous Brazilian studies is the true estimate.^25^

## RESULTS

### Patients

After linking the database entries from ANS, INCA/RHC, and FOSP, a total of 16,376 patients were identified. When screening for private health care patients, 5,217 patients were initially eligible. After a thorough review of the 5,217 patients, 199 had to be excluded because of inconsistencies (missing or no data) regarding treatment in the private setting. Thus, 5,016 patients with advanced NSCLC treated in private health care facilities were included in the analysis.

### Demographics

Most of the patients with NSCLC in the private setting were between 60 and 69 years old (33.6%) and had completed elementary school (52.2%) in the year of the diagnosis. A predominance of patients > 50 years age was observed in the sample, comprising more than 90% of the included patients. The assumption that the proportion of men with NSCLC should be greater than that of women was verified in Table 1 (58.1% *v* 41.9%), and the demographic homogeneity assumption was also verified below, given that key demographics were statistically similar throughout the years. A majority of patients had stage IV NSCLC (67%), and it was most often classified as adenocarcinoma (45.1%). The second most frequent subtype was squamous cell cancer (23.7%; Table 1).

**TABLE 1:**
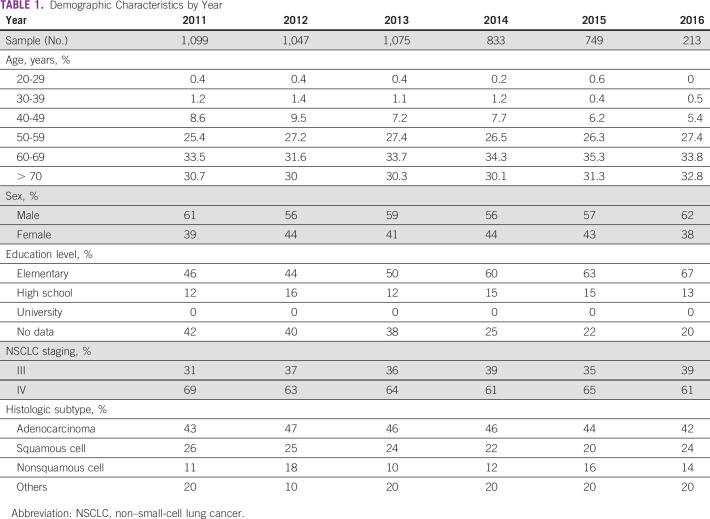
Demographic Characteristics by Year

### Access to Health Services and Resources Used from Diagnosis to Death

#### Access to health services.

The average time from first consultation to diagnosis was 31 days. In 44% of the cases, a clinical oncologist was the first health care professional that a patient was referred to, whereas in 21% and 17% of the cases, a patient was first consulted by a pulmonologist and thoracic surgeon. After the diagnosis, the median time to treatment start was 35 days (mean = 48 days).

#### Resources used: hospital stay.

If admitted to the hospital, patients with NSCLC stayed on average 7.4 days (ranging from 0 to 156 [min-max] days). On average, patients were admitted to the hospital twice (ranging from 1 to 15 [min-max] times). The median costs related to hospital admission were $466.66 USD ranging from $251.74 USD to $7,666.16 USD. The costs for the most common procedures or manipulations were as shown in Table 2—from the most used to less used: (1) chemotherapy for stage III or IV NSCLC ($420.04 USD [$32.13 USD-$2,347.45 USD]), (2) clinical complications in oncologic patients ($317.23 USD [$16.42 USD-$7,169.21 USD]), (3) brachytherapy ($1,064.26 USD [$392.67 USD-$4,319.37 USD]), (4) chemotherapy for stage III NSCLC ($999.52 USD [$392.67 USD-$2,356.02 USD]), and (5) chemotherapy for stage I to III NSCLC ($3,212.28 USD [$917.90 USD-$5,506.66 USD]).

**TABLE 2:**
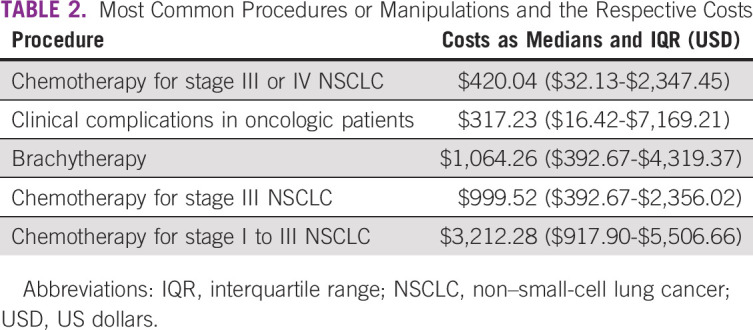
Most Common Procedures or Manipulations and the Respective Costs

#### Choice of therapy regimen.

The proportion of initial treatment regimen for patients with advanced NSCLC (with and without missing data) can be seen in Tables 3 and 4. The most frequent interventions are chemotherapy alone, followed by radiotherapy plus chemotherapy and radiotherapy alone.

**TABLE 3:**
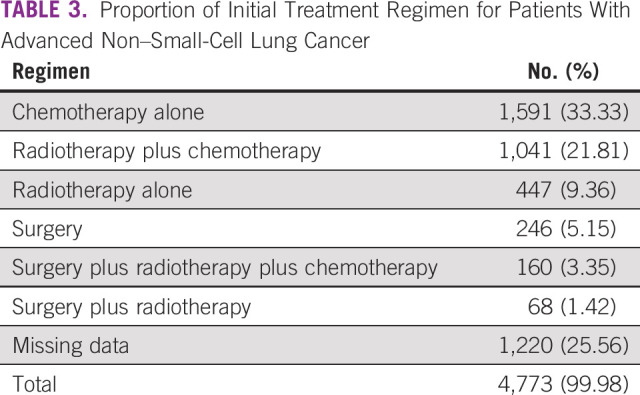
Proportion of Initial Treatment Regimen for Patients With Advanced Non–Small-Cell Lung Cancer

**TABLE 4:**
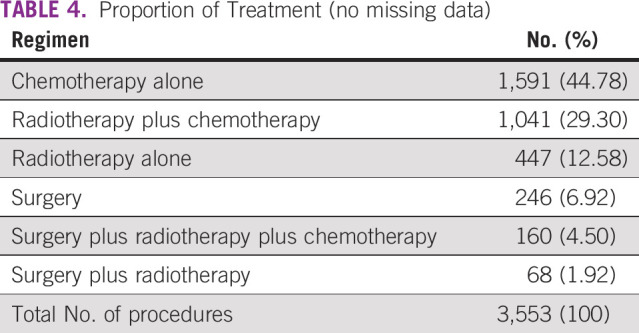
Proportion of Treatment (no missing data)

### Clinical Information and Outcomes (mortality)

#### Staging.

Of 5,016 patients in the studied sample, 1,346 (27%) were diagnosed with stage III disease, whereas stage IV accounted for 3,370 patients (67%). For this analysis, there were only 6% of missing data.

#### Survival.

**FIG 1:**
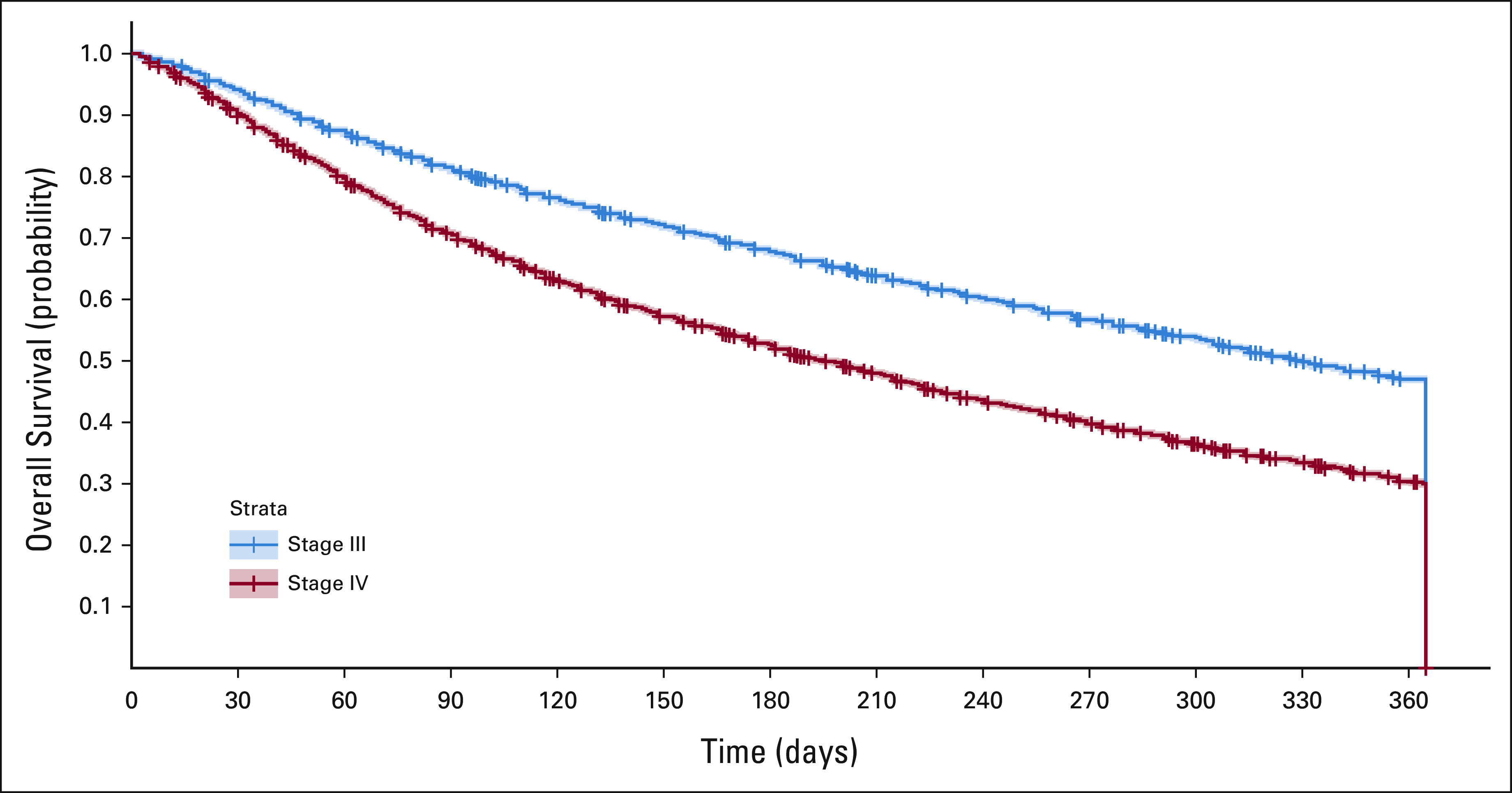
Kaplan-Meier estimates of overall survival for patients with stage III or IV NSCLC. NSCLC, non–small-cell lung cancer.

The obtained estimated median survival for the stage III patients was 11.5 months, whereas the median survival was 6.5 months for stage IV patients (Fig 1).

## DISCUSSION

The present study has the largest sample size ever published on patients with NSCLC treated in the private HCS in Brazil. It is also one of the largest cancer real-world data studies globally conducted in middle-income countries.^10,26,27^ Therefore, it brings a wider perspective on staging, survival, and resource use. This study allowed the elucidation of the survival profile of patients with advanced NSCLC in the private health care setting, which was comparable with survival rates in other countries, but from decades ago.^10,26,27^

Latin American health care database studies are scarce because of a lack of reliable and unified data sources. For NSCLC, real-world evidence is the ultimate modality of research to strategically assess the private health care market, especially when there is a lack of data published, as is the case with Brazil. The last review on lung cancer^10^ could not identify as much as 10 studies in the country. The sum of all patients in previous studies might be smaller than our sample size (eg, Barrios et al^25^ reported 41 patients). After matching the database records using the deterministic approach, we included more than 5,000 patients, which might not represent all patients with NSCLC who were referred to private health care institutions in Brazil, but certainly is the biggest source of information.

The predominance (> 90.0%) of patients > 50 years age in the sample was expected for patients with stage III and IV NSCLC.^10^ Regarding the assumption of a higher proportion of cases for men, the predominance of the disease in men was described here (58.1%) and confirmed elsewhere, varying from 2:1 to 12:1.^28,29^

Additionally, a possible explanation for the observed reduction in the number of NSCLC cases over time, in the private health care setting, includes different reasons: (1) One of the main databases used for cancer identification in Brazil (RHC/INCA) has not been locked (the data on the 2014-2016 cohort are still being collected). This observation was confirmed by the repeated download of databases on July 15, 2018, and September 20, 2018. (2) NSCLC definitions were changed in 2014-2015: pathologists were oriented to choose non–small-cell cancer, instead of non–small-cell lung cancer since the latter was confirmed after the exclusion diagnosis with or without identification of a pneumocyte marker to confirm a lung-specific disease.^30^ Per protocol, our mainstay criteria to identify patients with lung cancer were having ICD-10 code C34 and histologic findings compatible with NSCLC. Considering that malignant neoplasm not otherwise specified is a common diagnosis after the introduction of the 2014-2015 WHO Lung Cancer Classification, there was a significant loss of lung cancer registries.

Besides, our linkage method detected an underestimated but assertive number of NSCLC cases, which is preferable to overestimation. Considering the updated histologic classification from WHO, the current cancer registries might be subject to criticism on how accurate they are for estimating 34,000 new cases per year of lung or bronchus cancer in Brazil (public plus private systems).^10^ On the other hand, if the previous estimation is correct, the burden of NSCLC might be around 23,800 new cases per year (70% of all lung cancers, according to local experts^17^). Another relevant finding in the present study was access to health care services from the first consultation. We found an average 31-day interval to establish a diagnosis, which was corroborated by previous studies that suggested about 30 days to diagnose NSCLC.^29^ The median time to start treatment (35 days) was close to the findings from Abrao and Abreu,^31^ who suggested that the median time to start treatment was 1 month.^10^ In a sample of patients with stage III and IV disease, access to treatment in 30 days cannot be considered a standard of care and might affect mortality in Brazil. A later law, recently published (No. 13896/2019), stipulates that cancer treatment must start not later than 30 days after diagnosis. Although we have found that the timing is slightly above the legal limit, 30 days is still a long period to wait for a life-changing piece of news and it should be improved.^31^

Our analyses on resource use showed a profile similar to the private health care setting in Brazil, and these data could be used in the future as an important parameter for budget impact models.

Finally, the median survival for patients with stage IV NSCLC was 6.5 months, which is considerably lower than an American population cohort study that showed a median survival of 11 months.^32^ Additionally, a retrospective cohort study using data collected by the National Cancer Institute's SEER(from 1998 to 2003) Program described a median survival ranging from 4 to 6 months, according to the histologic subtype for patients diagnosed with stage IV NSCLC.^5^ The benchmark values for projecting survival analysis in Brazil are 8 and 7 months of life for stages III and IV, respectively.^10^ Variables that affect NSCLC survival rate, such as access to health services and the time to treatment start, might be considered before incorporating new technology. This has been associated with a decreased overall survival rate in studies conducted in Brazil and the United States.^33^ The introduction of new technologies, such as overcoming opportunities on immunobiology, which have been demonstrated to improve overall survival and progression-free survival compared with chemotherapy alone, is expected to improve the prognosis in the country. Nevertheless, knowing the survival rate, it is possible to design appropriate prospective studies and perform an economic analysis.^34^

Strengths and limitations may be found when working with secondary data, such as the databases used in this study. There is plenty of data to be analyzed, and, sometimes, they might be the only source available. On the other hand, the quality of the study will depend on the quality of the collected data and the format of their entry into the database may vary over time.

The core limitations of real-world data analysis include retrospective data collection, the administrative nature of the databases, and multiple individuals involved with data handling. To minimize the impact of the latter, we made sure that data were double-checked by different researchers. Our data might be confirmed by screening for logical associations (quality assurance of data, as we did by confirming logical assumptions, such as mortality, sex, and staging information).

In conclusion, this study provides contemporary real-world evidence on stage III and IV NSCLC in the private health care setting in Brazil and is the largest sample size observational study in lung cancer ever conducted in the country. The results show a high number of metastatic disease diagnoses, low survival rates, and high health care–related costs. The low survival rates are comparable with the pretargeted therapy era (median overall survival of 8-12 months). The reasons for the described high mortality should be investigated and discussed to improve the patients' survival and their quality of life. Timely access to more effective drug products must be sought to provide enhanced treatment and, therefore, improve the survival rates. Clearly, there is room for improving NSCLC care by stimulating earlier tumor detection and multidisciplinary approach and by providing access to molecular testing, chemotherapy, targeted therapies, and immunotherapies.
